# Contribution of Dendritic Cell Responses to Sepsis-Induced Immunosuppression and to Susceptibility to Secondary Pneumonia

**DOI:** 10.3389/fimmu.2018.02590

**Published:** 2018-11-13

**Authors:** Marwan Bouras, Karim Asehnoune, Antoine Roquilly

**Affiliations:** ^1^Surgical Intensive Care Unit, Hotel Dieu, University Hospital of Nantes, Nantes, France; ^2^EA3826 Thérapeutiques Anti-Infectieuses, Institut de Recherche en Santé 2 Nantes Biotech, Medical University of Nantes, Nantes, France

**Keywords:** dendritic cells, pneumonia, inflammation, immunity, innate, mucosal immunity, steroids, intensive care units

## Abstract

Dendritic cells (DCs) are bone marrow derived cells which continuously seed in peripheral tissue. During infection, DCs play an essential interface between innate and adaptive immunity. Pneumonia is a lung inflammation triggered by pathogens and is characterized by excessive release of inflammatory cytokines that activate innate and acquired immunity. Pneumonia induces a rapid and protracted state of susceptibility to secondary infection, a state so-called sepsis-induced immunosuppression. In this review, we focus on the role of DCs in the development of this state of immunosuppression. Early during inflammation, activated DCs are characterized by decreased capacity of antigen (cross)- presentation of newly encountered antigens and decreased production of immunogenic cytokines, and sepsis-induced immunosuppression is mainly explained by a depletion of immature DCs which had all become mature. At a later stage, newly formed respiratory immature DCs are locally programmed by an immunological scare left-over by inflammation to induce tolerance. Tolerogenic Blimp1+ DCs produce suppressive cytokines such as tumor growth factor-B and participate to the maintenance of a local tolerogenic environment notably characterized by accumulation of Treg cells. In mice, the restoration of the immunogenic functions of DCs restores the mucosal immune response to pathogens. In humans, the modulation of inflammation by glucocorticoid during sepsis or trauma preserves DC immunogenic functions and is associated with resistance to secondary pneumonia. Finally, we propose that the alterations of DCs during and after inflammation can be used as biomarkers of susceptibility to secondary pneumonia and are promising therapeutic targets to enhance outcomes of patients with secondary pneumonia.

Lung infection is a one of the main cause of mortality and morbidity worldwide (1). The overall death rate for patients with such infections was 2.6 million deaths worldwide in 2015, which is the leading infectious cause of death (2). However, the consequences of these infections cannot be reduced to the direct mortality from primary infection. Indeed, in critically ill patients recovering from a first severe sepsis (e.g., pneumonia or peritonitis), the risk for developing pneumonia reaches 30 to 50% (3) and in critically ill patients cured from primary pneumonia the early relapse with the same pathogen is up to 20%. (4). One of the main hypothesis to explain this susceptibility to infections is that patients with severe sepsis acquire a state of immunosuppression as evidenced by different host response during community-acquired and hospital-acquired pneumonia (5).

## Sepsis induced immunosuppression

Development of severe immune defects in immune-competent septic patients, a phenomena so-called “sepsis-induced immunosuppression” (6) has been associated with the risk of secondary pneumonia. During sepsis, the production and the release of pro-inflammatory cytokines is a necessary physiological phenomenon that activates the defense against bacterial infections and ensures injured tissue healing. To limit the risk of immunopathology observed during an overwhelming systemic inflammatory response syndrome (SIRS), whose main complication is a multi-organ failure syndrome (7), innate immunity cells rapidly develop a systemic compensatory anti-inflammatory response (CARS). This CARS aims to restore the state of immune homeostasis but either its prolongation or its exacerbation leads to an increased susceptibility to infections(6, 8).

So far, the main features of this sepsis-induced immunosuppression are

1. *A decreased antigen presentation ability by antigen presenting cells (APCs)*. APCs, mainly Dendritic Cells (DCs) and monocytes, have a central role in the capture, in the processing and in the presentation of antigens to effector lymphocyte T cells. These functions, essential for the establishment of an inflammatory response, are altered for weeks in mice and humans cured from systemic inflammation (9, 10)
2. *Dysregulation of the secretions of cytokines*. During infection, cytokines are messengers which ensure the coordination of all the cellular families. For example, APCs shape the response of effector T cells and innate-lymphoid cells to immunity or tolerance *via* the secretion of pro- or anti-inflammatory cytokines (e.g., Interleukin-12 or TGF-β). In critically ill patients, a decreased production of pro-inflammatory cytokines (such as TNF-α and IL-12) associated with a blunt release of anti-inflammatory cytokines (IL-10, TGF- β) have been associated with altered levels of pattern recognition receptors (11) epigenetic modifications (12) and post-transcriptional regulations.
3. *T cell exhaustion and apoptosis:* Exhaustion corresponds to the progressive loss of effector functions of T cells in the presence of a high antigenic load (13), while excessive inflammation results in caspase-3-dependent apoptosis (14, 15).

The capacity of DCs to detect environmental changes, to produce cytokines and present antigens to T cells suggests that they are a corner-stone of the physiopathology of the susceptibility to secondary pneumonia. Indeed, type 1 DCs (cDC1s) which are a highly potent cytokines secretion subtype of DCs, are a major source of IL-12 and hence promote NK and NKT cell IFN-γ production during systemic bacterial or viral infections (16). Mouse models of primary pneumonia (e.g., due to pneumococcal infection) have demonstrated a critical role for the activation of NK and iNKT in mediating the innate immune response to pulmonary infection (17) and especially in post-influenza bacterial secondary pneumonia (18, 19). In this review, we will thus focus on the fate of bona fide DCs (i.e., DCs not derived from monocytes) during and after sepsis, and will highlight the effects of glucocorticoids which are the first efficient immunotherapy in severe sepsis (20).

## Dendritic cells life-cycle before, during and after acute inflammation

Dendritic cells are bone marrow derived cells which play an essential interface between innate and adaptive immunity. DCs, which are the most potent antigen presenting cells (APCs), are involved in the initiation and the regulation of T cell-dependent immune response (21). According to the microenvironment and the signaling, DCs can secret pro-inflammatory cytokines to fight against infection or anti-inflammatory cytokines to maintain tolerance to self-tissue.

Before acute inflammation, DC precursors (pre-DCs) continuously leave the bone marrow as precursors and colonize peripheral tissues and lymphoid organs (e.g., spleen) where they develop into fully functional immature DCs (22). DCs are classified in different subsets: “plasmacytoid DCs” (pDCs) are the main source of type 1 interferons during many viral infections; the “conventional DCs” (cDCs), including mouse CD8+ cDCs and CD11b cDCs, have high antigen-presentation capacity and mainly produce other pro-inflammatory cytokines. In mice and human, two lineages of cDCs are clearly identified by differential expression of Xcr1 and Sirpa (23, 24) which recently allowed proposing a unified nomenclature of DCs across tissues and species, namely cDC1s and cDC2s, respectively (25). Indeed, the expression of CD141 (thrombomoduline) and CD1c (BDCA1) enable the distinction of two populations of Human DCs (26). The gene-expression profiles and functions of CD141+ cDCs and of CD1c+ cDCs resemble those of mouse cDC1 and cDC2 respectively (27, 28). cDC subsets are functionally well characterized: both cDC1s and cDC2s efficiently present extrinsic antigens on the MHC-II complex to CD4 T cells, although cDC2s appear to be more efficient for that function, cDC1s excel in antigen cross-presentation (presentation of extrinsic antigens to CD8 T cells on the MHC-I complex), although the other DC subsets can also exert this functions under specific conditions (29).

DCs can be further classified according to their organ localization and their migratory capacity: (1) the migratory DCs (including cDCs) are localized in peripheral tissues and migrate to the draining lymph nodes upon activation where they can exert their function of antigen presentation (for example Langerhans cells and dermal DCs), whereas (2) the resident DCs (including pDCs and cDCs) which remain in lymphoid organs where they locally collect Ag, (including from migratory DCs) to act as an amplificatory signal for T cell priming (for example thymic cDCs and splenic cDCs) (30). The classification proposed by Guilliams et al can integrate multiple layers of information in the denomination of DC subsets while still preserving a unifying nomenclature for their lineage belonging: for example, mouse spleen resident CD8a+ cDCs can be called “spleen resident CD8a+ cDC1s,” and the mouse CD103+ cDCs that have migrated from the skin into the cutaneous lymph node can be called “mouse CLN migratory CD103+ cDC1s.”

In steady state, the DCs have low expression of major histocompatibility complex class-II (MHC-II) and of membrane costimulatory molecules (such as CD86). DCs thus have high endocytic function for capturing Pathogen or Danger-associated molecular patterns (PAMPs or DAMPs), but are incompetent to present newly encountered antigens on MHC II molecules and to prime T-cells (31).

In the absence of infection, antigens presented by DCs silence effector T cells either by inducing apoptosis or by expanding regulatory T cells (32). This phenomenon has been recently better understood. In steady state, in contrast with DC maturation during inflammation, the maturation of migratory DCs (involving a novel NF-κB-regulated gene network) is associated with the induction of tolerance rather than T cell priming and activation (33). This process of terminal differentiation of steady state DCs is called “homeostatic maturation.” Some authors suggest that the signals triggering homeostatic, tolerogenic, DC maturation are conveyed via multiple pathways, some overlapping in part with those triggering inflammation but also leading to the expression of a specific transcriptional genetic program (34, 35). This homeostatic maturation leads to tolerogenic DC which promote the expansion of regulatory T cells (Treg) and tolerance to self-antigens (36).

During infection, the maturation of DCs is induced by the detection of PAMPs (*direct* activation) and by inflammatory cytokines released by other activated immune cells (*indirect* activation) (37). Direct activation of DCs induces several conformational and functional changes: (1) DCs become efficient at presenting the antigens by transient upregulation of MHC II synthesis (38); (2) they secrete cytokines for T cell polarization. Directly activated DCs are thus competent to prime naive T cells but they lose the ability to process and to present newly encountered antigens (9).

DCs can also be indirectly activated by inflammatory cytokines produced by PAMP-stimulated immune and epithelial cells (e.g., IFN-α/β, TNF-α …) (39). The levels of MHC-II and of co-stimulatory molecules are increased on the surface of indirectly activated DCs. Indirectly activated DCs can prime naive T cells like directly matured DCs, however their cytokine secretion function is altered and they retain the capacity to process new antigens (40, 41). During inflammation, directly and indirectly-activated DCs coexist and could theoretically be selectively targeted by interventions aiming to restore immune competence after inflammation.

**Figure 1:**
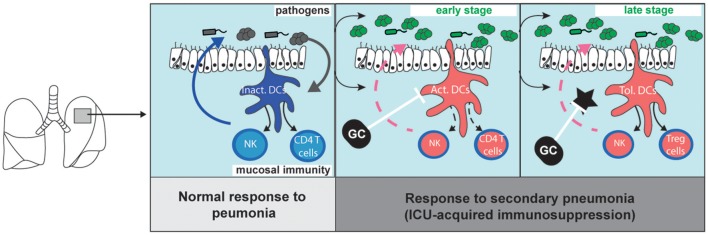
Migratory dendritic cell response during primary pneumonia, and during sepsis-induced immunosuppression (left). The stimulation of immature migratory dendritic cells (im.DCs) by pathogen-associated molecular patterns induces the production of inflammatory cytokines (such as Interleukin-12) which stimulate innate-like lymphocyte and natural killer cell (NK) functions and primes naive CD4 T cells. During sepsis-induced immunosuppression (middle and right panels), bacterial clearance is decreased as compared to what is observed during primary pneumonia. (middle) Early after primary infection, activated DCs (Act.DCs) are unable to respond to subsequent pathogens, and fail to produce cytokines and prime new CD4 T cells. (right) Lately, newly formed DCs locally acquire a tolerogenic programing (Tol. DCs) upon instruction by local tolerogenic mediators (star). Glucocorticoid (GC) inhibits DC activation and limit the SIRS. Upon stimulation by pathogens, Tol-DCs do not activate NK cells but induce the local accumulation of Treg cells. GC inhibits tolerogenic mediators and can restore immunogenic functions of newly formed DCs.

Protracted impairment of antigen presentation and of cytokine production in DCs of mice and patients cured from acute inflammation have been reported (42). Yet, new DCs continue to be produced after the onset of sepsis and inflammation with similar rates as in healthy conditions (43). Thus, after a few days, bodies cured from inflammation are seeded by newly formed immature DCs which are supposed to be fully functional. However, the susceptibility to infections last for weeks in critically ill patients cured from SIRS, and paralyzed DCs are still observed weeks after the cure from infections. If the paralysis of DCs lasts for weeks after inflammation, two periods can be distinguished: an early stage corresponding to the inflammatory response, and a later one lasting several weeks, probably months, after resolution of SIRS and characterized by an apparent return to non-inflammatory conditions but persistent dysfunctions of DCs. An important consideration when aiming to restore immune-competence during and after sepsis is to differentiate the mechanisms of DCs alterations during these two stages (Figure 1).

## Decreased number of immature DCs

The early decreased ability to present new antigens by the direct activation of DCs is not deleterious during local infections because a small number of DCs encounters the infecting pathogen and becomes activated, while the numerous remaining immature DCs can respond to new challenge. However, systemic circulation of PAMPs and of inflammatory mediators during sepsis causes systemic activation of DC, reducing the number of immature DCs capable of mounting an effective response to new threats, and limiting the ability of innate immunity to prime T cell responses (9, 44). The simultaneous activation of an excessive number of cDCs during systemic inflammation depletes the body from fully functional DCs and is thus immunosuppressive. Moreover, the total number of migratory and resident DCs is decreased following lung inflammation (45). The depletion of circulating DCs is reported in murine models of sepsis by caecal ligation and puncture (46) and the number of splenic DCs is decreased in patients dying from severe sepsis in intensive care units (15). Early after a lung infection by influenza virus, the presence of DCs in the lung was reduced (17, 47). Currently, the mechanisms of these “DC-penia” have not been fully elucidated. Some authors describe a defective *de novo* formation of DCs from common progenitors in the bone marrow (48) when others describe apoptotis mechanisms (46, 49, 50) or lysis by regulatory innate like lymphocytes (51). The mechanism involved in DCs apoptosis after SIRS is still unclear but a study has shown that an enzyme called acid sphingomyelinase (A-SMase), which is activated when DCs are treated with high numbers of *Escherichia coli*, induces apoptosis (52). The clearance of apoptotic DCs by viable DCs induces antigen-specific Tregs cells, and is thus probably beneficial to prevent auto-immune diseases (53). In addition to inducing immunosuppression by reducing the number of DCs, this phase of apoptosis could also induce a tolerogenic microenvironment maintaining this immunosuppressive state (54). The prolonged decrease in the number of circulating cDCs and pDCs has been associated with the risk of secondary infection in septic patients (55). This critical loss of DCs, which has also been associated with secondary pneumonia in burned patients (56) and in brain-injured patients (57), is probably a mechanism common to all the critical illness-inducing immunosuppression.

cDCs are continuously renewed from bone marrow pre-DCs and have a dependence for FLT3L/FLT3 (58). In the case of IAV infection, it seems that the drop of cDC number in the lungs is due to a defective FLT3L production (47). One the other hand, some DC-like cells, such as the mo-DCs (monocytes-derived DCs), are derived from monocytes in a GM-CSF dependent mechanism. In case of inflammation, an increase in the proportion of mo-DCs, which are more susceptible to polarization toward immunosuppressive functions by the local microenvironment, is also a cause of “sepsis induced immunosuppression.” Indeed, these mo-DCs have also been reported to induce TH2 and TH17 responses (59, 60). Sepsis-induced immunologic dysregulation occurs at every level of the ontogeny of each subset of DCs (61). Considering these results, several teams have hypothesized that the correction of the number of DCs after inflammation, notably by injecting FLT3L which is the DC growth factor, can restore immune-competence and limit the susceptibility to secondary pneumonia (47, 62, 63). To the best of our knowledge, the effects of FLT3L have never been investigated in septic patients, but GM-CSF, which is not specific to DCs but accelerates DC maturation, demonstrates disappointing effects in patients with sepsis (64, 65).

Patients lacking cDC2 due to IRF-8 genetic mutations are susceptible to infections (66). It is thus likely that lack of cDCs participates to the susceptibility to secondary infections, and functional defects of newly formed DCs can be of importance when aiming to restore a DC network after sepsis.

## Functional alterations of the newly formed DCs

Bone-marrow released pre-DCs reach peripheral tissue where they receive final differentiation messages and become fully functional. This final tissue maturation process explains the diversity of DC populations observed in the different organs in normal conditions and is called tissue-imprinting. It was recently shown after sepsis that the newly formed DCs are modulated both in the bone-marrow at a progenitor state (67) and locally in peripheral tissue at a final differentiation state by an immunological scare left-over by a primary inflammation response (10, 68). DC-precursors exposed to this new microenvironment are deficient for their capacity to produce IL-12, due to epigenetic alterations (69), impaired antigen (cross)-presentation capacity, and preferentially drive T cellular immunity to tolerogenic functions (10).

Several mediators of this suppressive-microenvironment left-over by primary sepsis have be demonstrated to be important as will be detailed below.

### Blimp-1

B lymphocyte-induced maturation protein-1 (Blimp-1) is a pleiotropic transcriptional factor which represses the IFN-β promoter and regulates functions of many immune cells, especially in lymphocytes (B and T cells). Blimp-1 is also expressed and functionally important for the myeloid lineage cells such as DCs and macrophages (70). The tolerogenic functions of Blimp-1 on DCs are well demonstrated in systemic autoimmune diseases, such as systemic lupus erythematosus. Mice with a Blimp-1ko phenotype in all CD11c-expressing cells including DCs (Blimp-1flox/flox; CD11c-CRE+) present an increased secretion of interleukin 6, an increased differentiation of effector T cells and suffer from the development of a lupus-like syndrome (71). Likewise, Blimp-1 regulates cDC2 homeostasis by preventing the excessive production of pro-inflammatory cytokines and overwhelming expansion of cDC2s after TLR stimulation (72). Blimp-1 could also be involved in SIRS and may be partly responsible for the observed susceptibility of patients to nosocomial pneumonia. We showed that cDC2s from patients suffering of SIRS expressed a high level of Blimp1 compared with healthy donors and thus lose their ability to produce type 1 cytokines (including interleukin-12) (10). Blimp-1 expression is also increased in DCs from patient suffering of post-trauma SIRS whose physiopathology is similar to sepsis and who are also susceptible to secondary pneumonia (73). In trauma patients, the increased expression of Blimp-1 has been correlated with the trauma severity (Glasgow Coma Scale) and with respiratory complications in intensive care unit (10). The overexpression of Blimp1 in cDC2s of critically ill patients recovering from a primary pneumonia might be a marker of the severity of immunosuppression and may thus allow identifying and treating early the patients at high risk of severe secondary infections.

### Interleukin (IL)-10

Numerous cell types, including NK cells, B cells, monocytes and DCs, were shown to produce IL-10 during “sepsis induced immunosuppression” (74).

IL-10 induces the apoptosis of mature DCs during chronic viral infections (75) and decreases the number of live DCs during post-traumatic pneumonia (76). In response to IL-12 secretion by mature DCs, NK cells rapidly express IL-10 which inhibits the production of IL-12 by DCs to prevent an overwhelming and deleterious immune response (51). For example, IL-10 neutralization by anti-IL-10 mAb restores the production of inflammatory cytokines, such as IL-12 and TNF-α, by DCs (77). During systemic infection, IL-10 inhibits the maturation of DCs and impairs the ability of cDC1s to prime a T cell response. This autocrine IL-10 regulation limits the development of new mature DCs (78) and limits the capacity of mature DCs to initiate Th1 responses. Immunosuppressive IL-10^+^ DCs induce Th2 response by stimulating cytokine secretion like IL-4 and “regulatory DCs” secreting IL-10 are also associated with up-regulation of T regulatory cells (T-reg). This regulatory mechanism is notably involved in hyper-eosinophilic airway inflammation (79, 80). IL-10 secretion is an essential component for the protective response against airway hyper reactivity and asthma (81) and is involved in development of lung tolerogenic DCs after pneumonia (82).

### Tumor growth factor-beta: TGF-β

TGF-β molecules act as cellular switches regulating numerous physiological processes such as immunity, cell renewal and healing. TGF-β is a pleiotropic cytokine involved in the development of Treg lymphocytes by inducing the *Foxp3* transcription factor expression in CD25^−^ naive T cells in order to enforce the transition to Treg cells (61). TGF-β are expressed constitutively by a wide variety of cells in the lung, including myeloid cells (DCs and alveolar macrophages), T cells and fibroblasts (83).

TGF-β are produced as inactive proprotein composed of mature TGF-β bound to latency-associated peptide. TGF-β activation from latency is controlled by numerous pathways that include actions of proteases present in the microenvironment such as plasmin, and/or by thrombospondin 1 or selected integrins expressed at the membrane of cDCs (84, 85). The unusual temporal discontinuity of TGF-β synthesis and action is an original mechanism which allows the TGF-β/LAP complex to behave as a matrix-localized sensor. During sepsis-induced immunosuppression, DCs are thus both a source and an activator of TGF-β in the tissue of mice cured from pneumonia (10). Our previous results indicate that cDCs of mice recovering from lung infection produce TGF-β and induce Treg cell accumulation (10). When they are activated by TGF-β after primary pneumonia, these Treg cell decrease the pro-inflammatory cytokine secretion pattern and the upregulation of CD80 and CD86 costimulatory molecules of immature cDCs, creating a tolerogenic environment (86). This mechanism is also found in intestinal epithelium where intestinal DCs promote a tolerogenic environment via TGF-β secretion to prevent an exacerbated response against the many non-pathogenic antigens in the gut (87). The crucial role of TGF-β in self-tolerance has long been established, with genetic deletion of TGF-β inducing multifocal inflammatory disease (88) or with the TGF-β down-regulation of co-stimulatory molecules expression on the surface of DCs limiting the functions of T cell effectors in the epidermis (89). The DCs-Treg cells-TGF-β loop plays a central role in the susceptibility to hospital-acquired pneumonia observed after severe infections.

## Glucocorticoids & dendritic cells

Lately after a primary lung inflammation, newly formed DCs receive tolerogenic messages during terminal differentiation in the tissue, and local imprinting drives DCs toward a new tolerogenic transcriptional programing (Figure 1). Tolerogenic DCs fail to develop immunogenic functions in response to subsequent infectious threats, and bacterial clearance is decreased during secondary pneumonia. Host-targeted approaches aiming to modulate the lung imprinting of DCs have the potential to restore immune competence after sepsis, and to decrease the risk of secondary pneumonia. Yet, specific interventions, such as the injection of blocking anti-IL-10 or anti-TGFβ antibody, have not been tested for the prevention of hospital-acquired infections in patients, probably because of safety concerns.

Glucocorticoids for the modulation of inflammatory-induced immunosuppression have been extensively tested in humans. Recent randomized studies have demonstrated that glucocorticoids decrease the risk of death of patients with septic shock (20) or with community acquired pneumonia (90). Low doses of steroid also prevent hospital-acquired pneumonia in severe trauma patients (91). It can seem counterintuitive to use drugs classically considered as immunosuppressive in patients with severe infections or at high risk of sepsis. Indeed, glucocorticoids are highly anti-inflammatory molecules (92) and steroids have long been indicated for the management of patients suffering from non-septical inflammatory diseases such as rheumatoid arthritis or systemic erythematous lupus (93), and for the induction of tolerance to graft (94). A reappraisal of the immunological effects of steroids during acute inflammation, and a better comprehension of the impacts of inflammation on the development of immune response to secondary infections, have provided the rational to explain these clinical observations. We propose that steroids prevent the excessive activation of DCs during the severe inflammatory stage (Figure 1, middle panel) and limit the alterations of DCs observed during the late stage of sepsis-induced immunosuppression (Figure 1, right panel).

It has been long known that the Hypothalamic-Pituitary Adrenal (HPA) axis, and in particular glucocorticoids, is a major component of the response to sepsis (95), as demonstrated by the susceptibility of adrenalectomized mice to septic shock (96). Endogenous glucocorticoid (i.e., cortisol), as well as therapeutic glucocorticoids (i.e., dexamethasone), control many essential metabolic, cardiovascular, and homeostatic functions during inflammation. These numerous effects results from the pleiotropic activity of the glucocorticoid receptor (GR) on multiple gene promotors and on multiple target cells (94). Multiple GR isoforms exist (including the main GRα and β receptors) with distinct tissue distribution patterns and functions. The activated glucocorticoid–glucocorticoid receptor-alpha (GC-GRα) complex acts at the intra-cytoplasmic level to reduce the post-transcriptional expression of pro-inflammatory cytokines and to increase the transcription of anti-inflammatory and tolerogenic genes (94).

Endogenous or synthetic glucocorticoids particularly influence the innate immune cells during the inflammation period. One of the main targets of glucocorticoids are innate lymphoid cells and the neuroendocrine axis is crucial for tolerization of the innate immune system to microbial endotoxin exposure through direct corticosterone-mediated effects on innate cells (97). Glucocorticoids also modulate DCs during and after inflammation (98). *In vitro*, exogenous GCs at therapeutic concentrations inhibit the differentiation of DCs from their precursor cell (99), and limit their activation by PAMPs/DAMPs (100). GCs induce apoptosis of mature migratory DCs *in vivo* and *in vitro* (101). Interestingly, many studies have demonstrated that glucocorticoids suppress mature DCs but spare immature DCs *via* a differential expression of GR translational isoforms (102, 103) and the activation of cell survival pathways (104). Endogenous glucocorticoid elevation following pneumonia participates to the clearance of mature pro-inflammatory cDCs and to the development of tolerogenic DCs (105–107). In humans suffering from septic shock, GC restores MHC-II expression on myeloid cells, suggesting a better antigen presentation by APCs during treatment (108). During viral pneumonia, the initial hypercorticism limits the inflammatory-induced lung injuries and prevents mortality during bacterial superinfection (109). This protective effect (108) is notably mediated by direct effect of GC on the cytokine production by DCs since the conditional deletion of GR in CD11c+ cells prevents mice from death upon LPS stimulation. These results suggest that glucocorticoids are necessary to control the initial inflammatory response, limiting the initial shortage on immature DCs, and limiting the local imprinting which induces the formation of tolerogenic DCs for weeks after the primary pneumonia.

## Conclusion

Clinical and bacterial cure failures are common in patients treated for pneumonia, and the susceptibility to secondary infection is high. These observations have been linked to the development of sepsis-induced immunosuppression. Acquired alterations in the numbers and functions of respiratory DCs are crucial in this condition. To develop targeted-host approaches, it is necessary to closely consider the timing of the interventions. A loss of immature DCs is the main mechanisms during the early phase, and alterations of the terminal maturation of newly formed DCs participate to the month-long susceptibility to secondary pneumonia. To treat the sepsis-induced immunosuppression, and limit the susceptibility to secondary pneumonia, many therapies have been tested in recent years. They aimed either to limit the initial SIRS (and thus the CARS) in particular by the use of low dose glucocorticoids (20, 91, 110) or to restore or supplement the secretion of pro-inflammatory cytokines by the injection of IFN-γ, GM-CSF (110) or interleukin-12 (10).

Using exogenous glucocorticoid at early phase of sepsis may limit the immune paralysis by decreasing the number of tolerogenic mature DCs and by limiting the development of a tolerogenic trained innate immunity.

## Author contributions

MB and AR wrote the draft. KA extensively reviewed the manuscript. All the authors approved the manuscript before the submission

### Conflict of interest statement

The authors declare that the research was conducted in the absence of any commercial or financial relationships that could be construed as a potential conflict of interest.
